# Genetic editing and interrogation with Cpf1 and caged truncated pre-tRNA-like crRNA in mammalian cells

**DOI:** 10.1038/s41421-018-0035-0

**Published:** 2018-07-10

**Authors:** Xuhua Zhang, Linping Xu, Ruihua Fan, Quanli Gao, Yunfeng Song, Xiaodong Lyu, Jiangtao Ren, Yongping Song

**Affiliations:** 1 School of Life Sciences, Zhengzhou University, Zhengzhou, China; 2 Affiliated Cancer Hospital of Zhengzhou University, Henan Cancer Hospital, Zhengzhou, China; 3 Bioheng Technology Inc., Nanjing, China

## Abstract

Cpf1, an RNA-guided DNA endonuclease that belongs to a new class II CRISPR system, has recently been harnessed for genome editing. Herein, we report an RNase-resistant caged truncated pre-tRNA-like crRNA (catRNA) that confers precise and efficient gene editing with the *Lachnospiraceae bacterium* Cpf1 (LbCpf1) and enables the reprogramming of catalytically dead LbCpf1 (dCpf1) lacking DNA endonuclease activity into a transcriptional modulator. Specific gene knock-outs and knock-ins were increased 3.2-fold and 4.3-fold, respectively, with catRNA compared to that induced by conventional crRNA. A much higher augmentation of gene disruption (up to 37-fold) was observed when electroporation was used. We report herein that catRNA enables efficient gene activation with dCpf1 activators. Our study reveals the potential of catRNA and a versatile application of the CRISPR/Cpf1 system, establishing a simple approach for selective gene perturbation in mammalian cells.

## Introduction

Cpf1 (CRISPR from *Prevotella* and *Francisella* 1), recently characterized as a novel class II CRISPR system component that has features distinct from those of Cas9, is a single RNA-guided endonuclease that recognizes thymidine-rich protospacer adjacent motifs (PAMs) and produces staggered cuts distal to the PAM site^1^. This type V CRISPR/Cpf1 system has demonstrated robust genome editing activity in mammalian cells^1,2^ as well as in animals^3^, plants^4–8^, and bacteria^9,10^. Interestingly, Cpf1 is a dual nuclease that not only cleaves target DNA but also processes its own CRISPR RNA (crRNA)^1,11^. In addition, the maturation of crRNA by Cpf1 does not require assistance from trans-activating crRNA (tracrRNA). Harboring these advantages, the Cpf1 system was recently adopted for multiplex gene editing in mammalian and plant cells, wherein up to four genes were simultaneously edited by Cpf1 using a single crRNA array spaced by mature direct repeats^12,13^.

Compared with the high off-target potential of Cas9, genome-wide deep sequencing revealed very precise gene disruption by Cpf1^2,14^, making Cpf1 an ideal gene modification alternative to Cas9.

Although Cpf1 mediates efficient gene editing in mammalian cells, its overall activity is not as robust as that of Cas9^1,2,14^. We speculated that the crRNA partially underlies its reduced gene disruption efficiency, as fewer stem loop structures present in crRNA confer less RNase resistance than that provided by various stem loops in single-guide RNAs (sgRNAs)^15–17^. Transfer RNAs (tRNAs) are the most stable RNA molecule because they harbor multiple hairpins (or stem loops) and 3′ trailer tail structures that impart resistance to various RNase molecules. Precursor-tRNAs (pre-tRNAs) are occasionally capped with methylguanosine at their 5′ termini^18^. A recent study in *Saccharomyces cerevisiae* found that methylguanosine cap structures protect pre-tRNAs from degradation by RNases, and the cap structures likely act as a shield to protect pre-tRNA during the maturation process^19^. We engineered an RNase-resistant caged truncated pre-tRNA-like crRNA (catRNA) with a 5′ cap and a 3′ tail, demonstrating that robust gene editing in mammalian cells can be achieved.

Despite the known application of Cpf1 to generate genomic mutagenesis by inducing double-stranded breaks, its potential as a transcriptional modulator for gene interrogation has not been fully elucidated. A recent study in *Arabidopsis* demonstrated for the first time that endogenous miR159b expression in plants can be sharply reduced by fusing an SRDX repressor domain to the catalytically dead LbCpf1 (lacking DNase endonuclease activity) and *Acidaminococcus* Cpf1 (AsCpf1)^20^. Another study demonstrated that DNase-deactivated Cpf1 from *Eubacterium eligens* (EedCpf1) can be reprogrammed as an efficient gene repression platform in bacteria^21^. *Francisella tularensis* Cpf1 (FnCpf1) was also adopted for multiplex gene regulation in *Escherichia coli*^22^.

To test the feasibility of genetic interrogation in mammalian cells by reprogramming Cpf1 into a transcriptional modulator, we fused VP64 and VPR activator domains to catalytically dead LbCpf1 (dCpf1). We demonstrated that compared to conventional crRNA, catRNA enabled better gene interrogation. Harnessing this feature, we achieved efficient genetic perturbation in mammalian cells with dCpf1 activators.

## Results

### Efficient gene ablation with caged truncated pre-tRNA-like crRNA

**Fig. 1:**
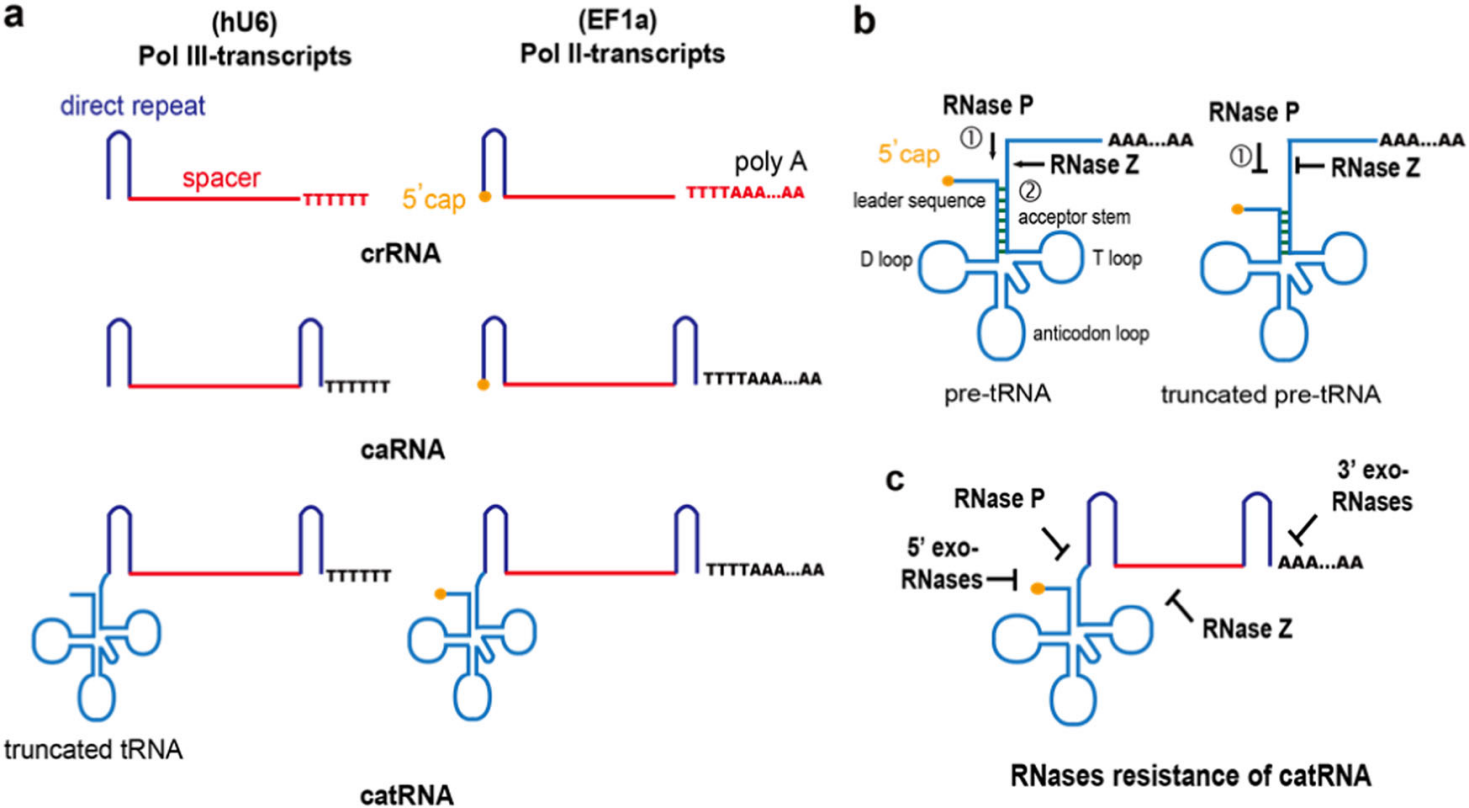
Design of RNase-resistant caged truncated pre-tRNA-like crRNA (catRNA). **a** Structures of conventional crRNA, caged crRNA (caRNA), and caged truncated pre-tRNA-like crRNA (catRNA) transcribed by Pol III and Pol II polymerases. Red, crRNA spacer sequence. Oligo-T (Pol III) or oligo-T-Poly A (Pol II) are incorporated into crRNA but not caRNA or catRNA. **b** Structures of pre-tRNA and truncated pre-tRNA. Pre-tRNA is matured by RNase P by removal of the leader sequence and subsequently modified and processed by RNase Z. RNase P processing is blocked by disrupting the 7-base pair stem structure of pre-tRNA, which is critical for RNase P recognition and cleavage. Subsequent RNase Z cleavage is also abolished due to RNase P processing failure. **c** Illustration of the RNase-resistant property of catRNA

**Fig. 2:**
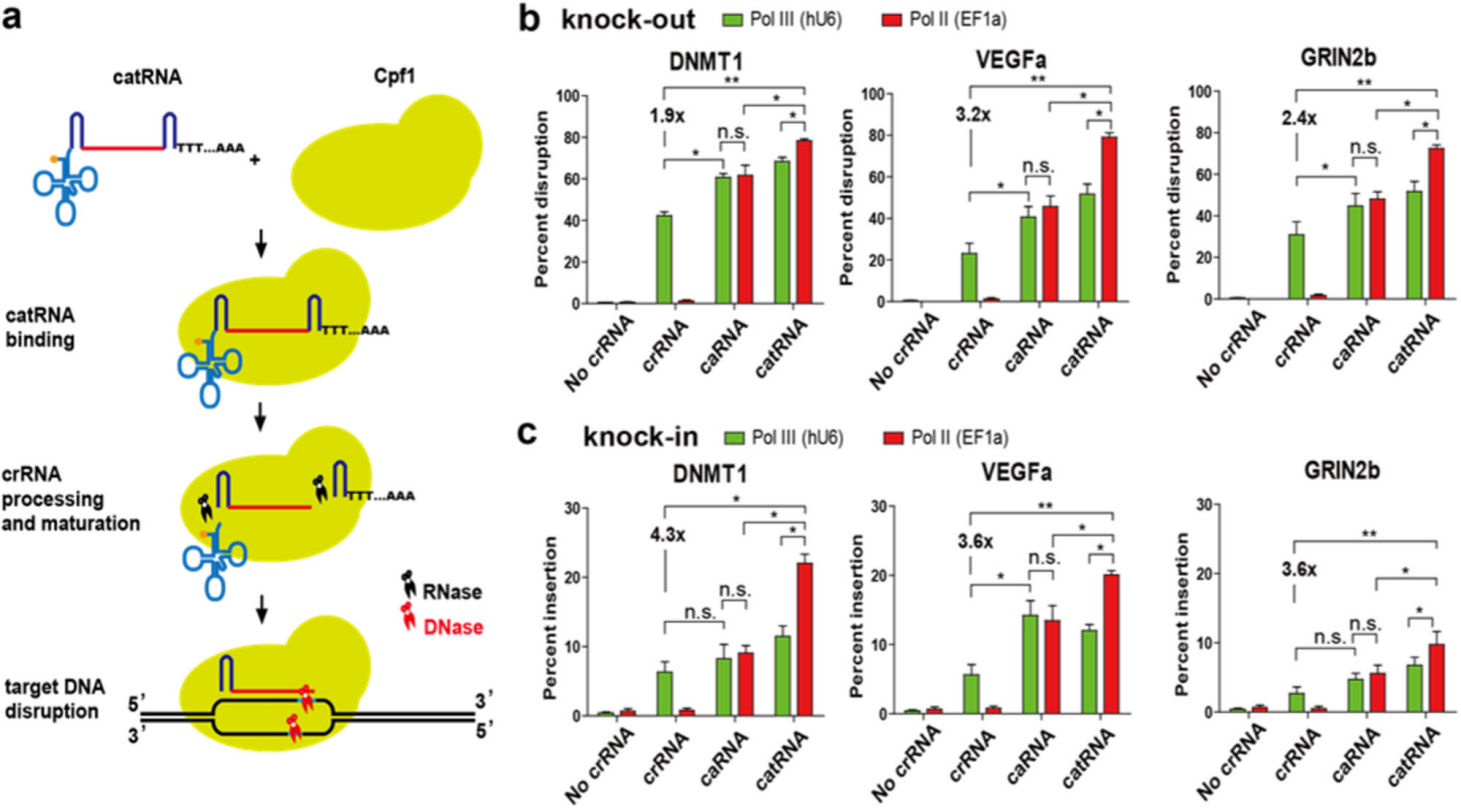
catRNA enhances knock-out and knock-in in mammalian cells. **a** Schematic flowchart of the catRNA maturation process and subsequent gene disruption. **b** Targeted gene knock-out with crRNA, caRNA, and catRNA in 293T cells. Gene knock-out was performed by co-transfecting the LbCpf1 DNA plasmid and plasmids encoding different crRNA species with Lipofectamine 3000. Specific gene knock-outs and knock-ins were measured 3 days after transfection with TIDE online software. **c** Targeted knock-in with crRNA, caRNA, and catRNA in 293T cells. Knock-in was performed with LbCpf1 and different crRNA species together with ssDNA donor templates. An additive effect was always observed when the 5′ cap, 3′ tail, and truncated tRNA structures were used in combination in knock-outs and knock-ins with catRNA. Specific gene knock-outs and knock-ins were measured 3 days after transfection with TIDE online software. Bar, SE. *n* = 3. *P < 0.05, **P < 0.01, ***P < 0.001, ****P < 0.0001 determined by the Mann–Whitney test

To test whether CRISPR/Cpf1 could efficiently disrupt genes in mammalian cells, we co-transfected an LbCpf1 plasmid with plasmids carrying crRNAs targeting the DNMT1, VEGFa, and GRIN2b genes driven by a U6 polymerase III (Pol III) promoter in 293T cells. However, after initial testing, only moderate gene disruption was detected. We reasoned that U6 Pol III-transcribed crRNA (Pol III-crRNA) products contain 3′ terminal oligo U sequences, which can be incorporated into spacer crRNA sequences and might affect target DNA recognition by the crRNA (Fig. 1a). We in vitro transcribed conventional crRNA and crRNA with different 3′ terminal oligo U sequences (oU-crRNAs) targeting the DNMT1 and VEGFa genes by T7 polymerase. We compared the gene disruption efficiency of crRNA and oU-crRNA by electroporating equal amounts of crRNA and oU-crRNA after Cpf1 mRNA electroporation into 293T cells. In both genes, crRNAs exhibited higher gene targeting efficiency than oU-crRNAs (Supplementary Fig. 4). This result indicates that an additional nucleotide at the 3′ end of crRNA that does not match the target sequences reduces the targeting efficiency. A similar phenomenon was observed when an extra mismatched nucleotide was added to the 5′ end of Cas9 sgRNA, especially high-fidelity Cas9 variants^23^. To solve this problem, we generated a caged crRNA (caRNA) with a spacer sequence flanked by two direct repeat sequences (Fig. 1a). As Cpf1 also cleaves pre-crRNAs upstream of hairpin structures formed within CRISPR repeats, functional crRNA is not released from caRNA by Cpf1 until the extra direct repeat is cleaved (Fig. 2a). We found that caRNAs efficiently disrupted DNMT1, VEGFa, and GRIN2b with efficiencies reaching 60.7, 47.3, and 38.0%, respectively; the efficiencies of disrupting these genes with crRNAs were only 42.1, 23.2, and 32.1%, respectively (Fig. 2b).

tRNAs are the most stable RNA molecules because they harbor multiple hairpins and 3′ trailer tail structures that render them resistant to various RNases. A recent study demonstrated that occasional pre-tRNA 5′ capping protects pre-tRNAs from RNases^19^. Pre-tRNA is processed into mature tRNA by removal of the 5′ leader sequence with RNase P followed by RNase Z cleavage at the 3′ end (Fig. 1b). As the 7–9-base pair acceptor stem structure is critical for RNase P recognition and cleavage, disruption of the acceptor stem will limit leader cleavage and tRNA maturation by RNase P^24,25^. In addition, because RNase Z accepts only tRNA precursors with mature 5′ ends, hindering RNase P processing will also cause RNase Z cleavage failure^26^.

To further improve the caRNA stability, we utilized pre-tRNA to make a caged, RNase P-resistant, acceptor stem truncated, 5-base pair stem pre-tRNA-like crRNA driven by an EF1a Pol II (Pol II-catRNA) promoter rather than the U6 Pol III promoter to ensure methylguanosine capping of catRNA. This catRNA is also exempt from RNase Z processing due to the absence of RNase P processing and RNA editing. As the catRNA 5′ cap and 3′ tail also provide protection against exo-RNases and mature crRNA is not exposed to RNases until Cpf1 processing, we hypothesized that catRNA possesses a much longer half-life than crRNA and caRNA and might enhance gene editing. Very interestingly, the efficiencies of targeting DNMT1, VEGFa, and GRIN2b with Pol II-catRNA, but not with Pol III-catRNA, were further improved 1.9-fold, 3.4-fold, and 2.4-fold, reaching efficiencies of 78.3, 79.2, and 76.8%, respectively. The results were confirmed by TIDE analysis and T7E1 assays (Fig. 2b and Supplementary Fig. 5). This result suggests that Pol-II catRNA provides a substantial gene editing benefit. While insufficient processing of Pol II-crRNA entirely abolished its function, Pol II-caRNA resulted in gene ablation similar to that induced by Pol III-caRNA but did not further improve the disruption efficiency, indicating that the 5′ cap alone did not augment gene ablation (Fig. 2b).

### Additive effect of caged truncated pre-tRNA-like crRNA elements

To confirm that the increased gene disruption was due to enhanced catRNA stability and not differential transcription by Pol II and Pol III, we electroporated equal amounts of in vitro-transcribed DNMT1-specific Pol III transcript-like unmodified crRNA, caRNA, and catRNA and Pol II transcript-like 5′ capped-3′ tailed crRNA, caRNA, and catRNA into 293T cells and measured their persistence by quantitative real-time PCR. Consistent with our hypothesis, only one of the RNAs was detectable 30 min after electroporation, Pol II-catRNA, which was even still detectable 4 h after electroporation (Supplementary Fig. 1a). Enhanced stability of Pol II-catRNA was also confirmed by in vitro RNase A digestion assays (Supplementary Fig. 3).

A recent study by Zhong et al. showed that Pol II-transcribed functional crRNAs, which resemble Pol-II caRNAs in this study, exhibit higher gene targeting efficiency than Pol III-transcribed crRNAs^27^. We also observed a trend toward higher efficiency using Pol-II caRNA; however, the levels of efficiency were not comparable to those of catRNA in our experiment (Fig. 2b). To test whether the higher gene disruption was due to the protective effect of transcripts upstream of the catRNA sequences, we embedded caRNA into the 3′ UTR of GFP mRNA to mimic the Pol-II crRNA described by Zhong et al. (m-caRNA). However, significantly augmented gene disruption was observed only when Pol II-transcribed RNase-resistant catRNA, rather than crRNA, caRNA, or m-caRNA, was used. m-caRNA exhibited a better gene disruption efficiency than Pol-III caRNA but had an ability similar to that of Pol-II caRNA (Fig. 2b and Supplementary Fig. 2a). These data indicate that the protective effect of catRNA arises from multiple hairpin structures in truncated tRNA rather than from an mRNA transcript that is more susceptible to RNase degradation.

We reasoned that an additive effect exists when using a truncated tRNA structure, 5′ cap and 3′ tail in combination, as neither the 5′ capped-3′ tailed Pol II-caRNA nor the truncated tRNA-like Pol III-catRNA conferred enhanced RNase-resistant stability. Previous studies demonstrated that tRNA-flanked sgRNAs and crRNAs can be accommodated for efficient multiplex genome editing^28,29^. To further confirm that the protective ability arises from the tRNA hairpin structure itself and not from the RNase-resistant property of 5′ truncated tRNA, full-length tRNA with an intact acceptor stem was tethered to caRNA. However, no detectable enhancement was observed with this construct (Supplementary Fig. 1b). This result was expected since tRNA is released from the pre-tRNA–crRNA complex by RNase P and RNase Z immediately after electroporation into 293T cells and no longer provides protection for crRNA. Taken together, these data suggest that the 5′ cap, 3′ tail, and 5′ truncated hairpins in combination are crucial for the increased stability of caged pre-tRNA-like crRNA.

### catRNA boosts homologous recombination-mediated targeted gene knock-in

To test whether catRNA enhances site-specific knock-in, we co-transfected plasmids encoding LbCpf1 and different crRNA species together with a single-stranded DNA (ssDNA) donor template. Pol II-catRNA greatly enhanced homologous recombination, as targeted knock-ins with catRNAs at DNMT1, VEGFa, and GRIN2b loci were increased 4.3-fold, 3.6-fold, and 3.6-fold, respectively, compared with those achieved using conventional crRNA. Although caRNA exhibited significantly elevated knock-in at only the VEGFa locus, slight increases were also observed at DNMT1 and GRIN2b loci (Fig. 2c). No significant differences in the knock-in abilities of Pol-II and Pol-III caRNAs were observed at any of the 3 target sites, but Pol-II catRNA increased knock-in at the 3 target sites much more efficently than Pol-III catRNA (Fig. 2c and Supplementary Fig. 6).

As routinely observed in the CRISPR/Cas system, the efficiency of knock-in is always lower than knock-out as NHEJ is more frequently used by DNA repair pathway than homologous recombination. Because homologous recombination occurs after double-stand break generation, the higher the cutting efficiency, the higher the chances of homologous recombination.

### catRNA enhances the efficiency of targeted gene knock-out with RNA transfection

**Fig. 3:**
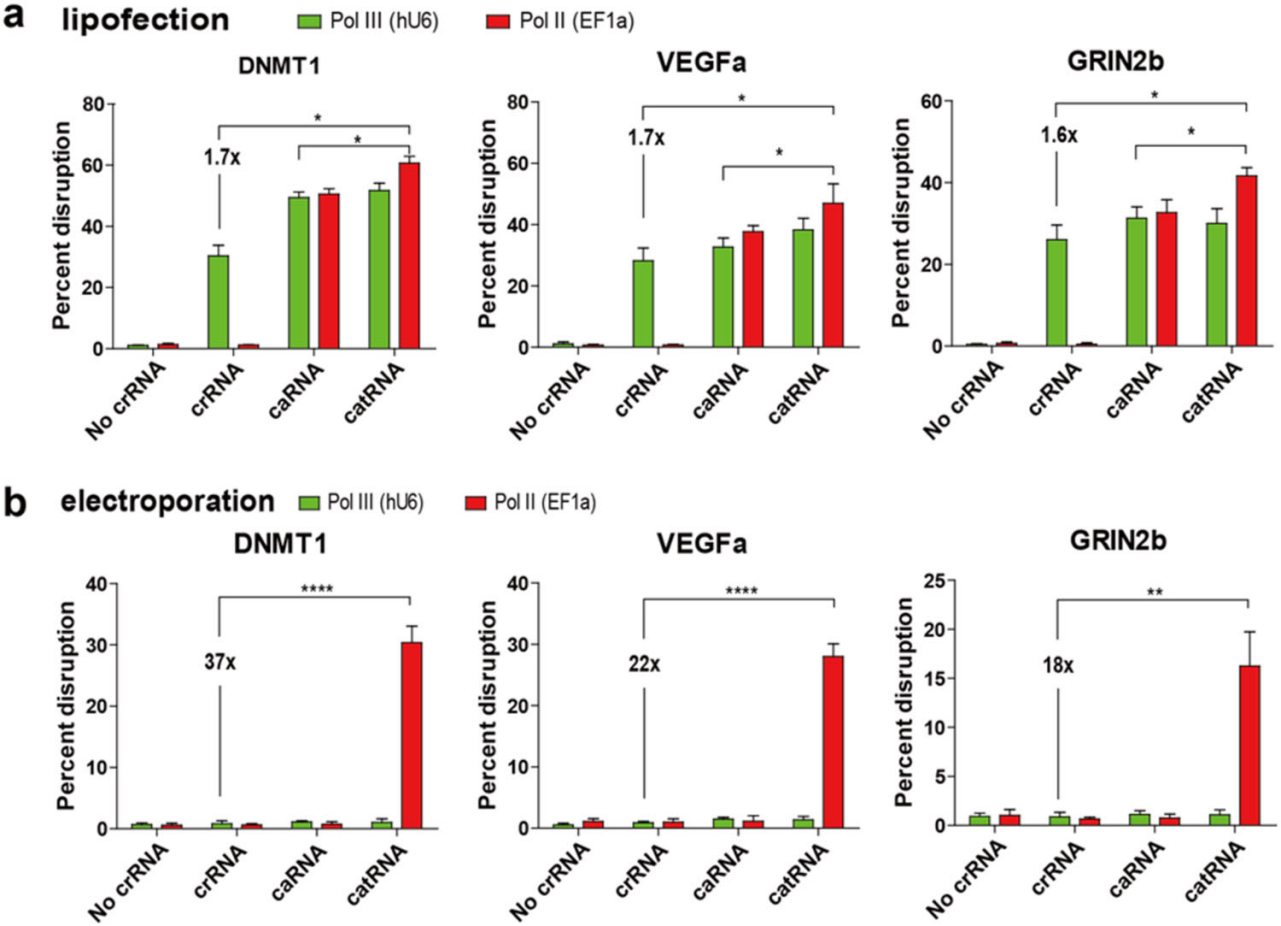
catRNA enhances targeted gene knock-out with RNA transfection. **a** Targeted gene disruption with different crRNA species by RNA lipofection. LbCpf1 mRNA was produced via in vitro RNA transcription and then co-transfected with different crRNA species using lipofection. Significantly enhanced gene disruption was consistently observed with catRNA transfections. *n* = 6. **b** Targeted gene disruption with different crRNA species by RNA electroporation. LbCpf1 mRNA was co-electroporated with different crRNA species. Robust gene disruption was observed with catRNA electroporation but not with electroporation of other crRNA species. Gene disruption efficiency was calculated 3 days after transfection by TIDE online software. Bar, SE. *n* = 3. *P < 0.05, **P < 0.01, ***P < 0.001, ****P < 0.0001 determined by the Mann–Whitney test

To test whether catRNA enhances RNA transfection-mediated gene editing, we performed gene disruption with in vitro-transcribed LbCpf1 mRNA and crRNA by liposomal transfection in 293T cells. Notably, the catRNA-induced gene disruptions of DNMT1, VEGFa, and GRIN2b were 1.7-fold, 1.7-fold, and 1.6-fold higher, respectively, than those achieved with crRNA. Substantially elevated gene disruptions were obtained at the DNMT1, VEGFa, and GRIN2b loci when catRNA was used compared to that achieved by caRNA. In addition, Pol II-catRNA constantly showed better gene disruption than Pol-III catRNA (Fig. 3a).

Gene editing efficiency with RNA transfection methods heavily relies on the delivery method. Compared to lipofection, which delays the release of RNA to cytosolic RNases because of an endocytosis process that allows time for Cpf1 protein synthesis and complex formation with crRNA, electroporation directly exposes RNA to cytosolic RNases and renders crRNA susceptible to degradation.

To test whether the RNase-resistant property of catRNA enhances gene editing efficiency via electroporation, we electroporated Cpf1 mRNA together with different crRNA species directly into 293T cells. Substantially elevated gene disruption was observed with electroporation of Pol II-catRNA but not with electroporation of Pol-III-catRNA or other crRNA species. Marginal (0.8–1.3%) gene disruption was observed with crRNA and caRNA, while we observed 37-fold, 22-fold, and 18-fold increases (30.3, 24, and 16.1%) in gene disruption at the DNMT1, VEGFa, and GRIN2b loci, respectively, with Pol II-catRNA but not Pol III-catRNA, probably due to the protection provided by the 5′ cap and 3′ Poly-A tail of the Pol II transcript (Fig. 3b). We also failed to detect any gene disruption when the full-length pre-tRNA-crRNA, 5′ cap-3′ tail Pol II-caRNA, or truncated tRNA-like Pol III-catRNA were used, further confirming the critical requirement of the entire truncated pre-tRNA-crRNA chimera structure for gene editing (Supplementary Fig. 1c). Therefore, we believe that catRNA will be very useful in fields wherein RNA electroporation is less toxic and favorable, such as gene editing in embryos and immune cells.

### catRNA enables efficient gene activation with dCpf1 activators

**Fig. 4:**
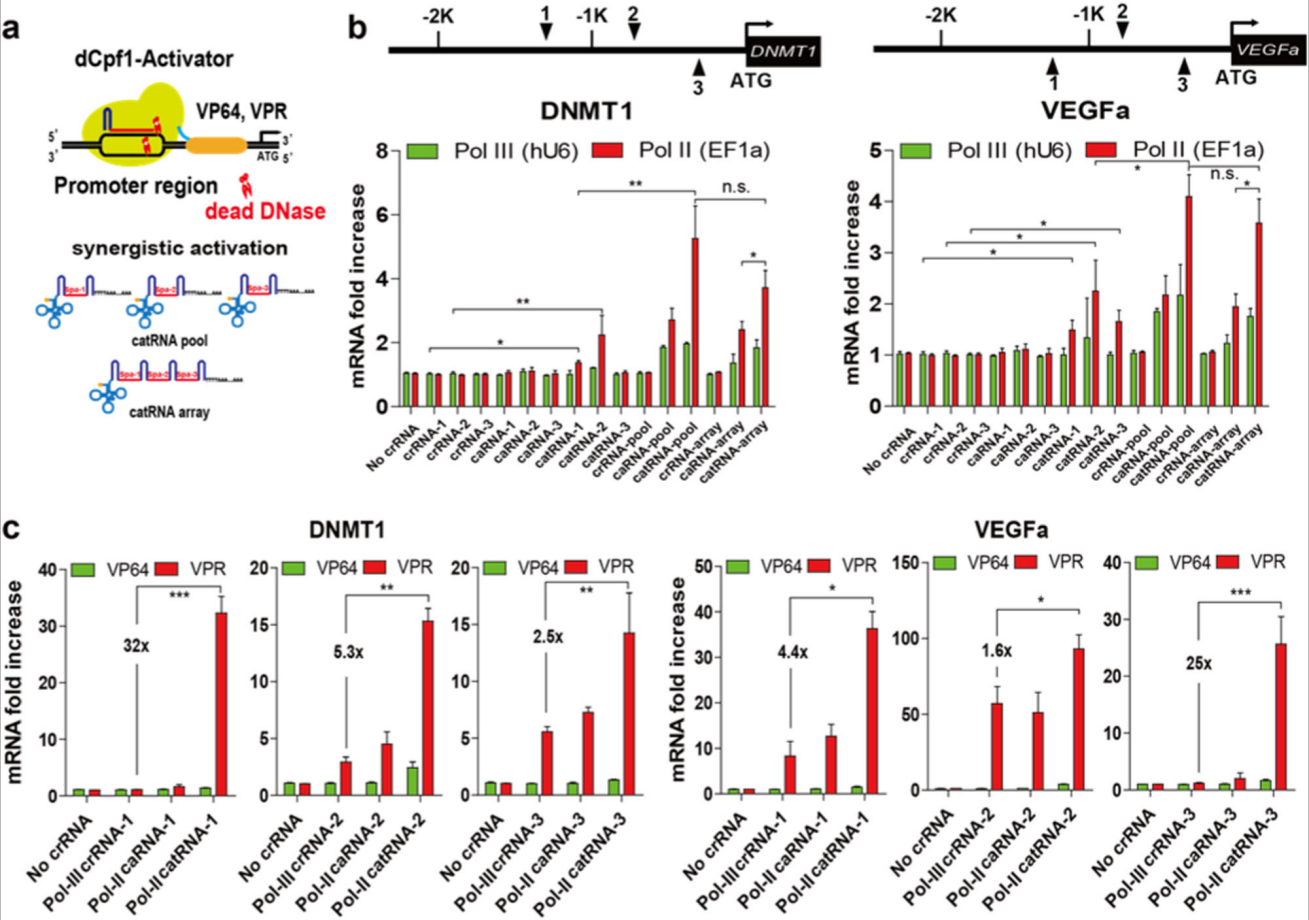
catRNA enables efficient gene activation with catalytically dead LbCpf1 activators. **a** Schematic representation of using the dCpf1-VP64/VPR activator and crRNA/catRNA arrays for synergistic gene activation. Spa: Spacer. **b** Synergistic gene activation with the dCpf1-VP64 activator. Plasmids encoding single or multiple crRNAs or a crRNA array targeting the DNMT1 and VEGFa promoter regions were transfected together with the dCpf1-VP64 plasmid into 293T cells. Gene expression was measured by quantitative real-time PCR 48 h post-transfection. **c** Efficient gene activation was achieved with a single catRNA and the dCpf1-VPR activator. Single crRNA, caRNA, or catRNA targeting the DNMT1 and VEGFa promoter regions were co-transfected with the dCpf1-VPR fusion protein into 293T cells, and gene expression was calculated by quantitative real-time PCR 48 h post-transfection. Bar, SE. *n* = 3. *P < 0.05, **P < 0.01, ***P < 0.001, ****P < 0.0001 determined by the Mann–Whitney test

While Cpf1 has been utilized for gene disruption, gene regulation with catalytically dead LbCpf1 (dCpf1) is not well studied. To implement dCpf1 in gene activation, we tethered four copies of the herpes simplex virus-derived VP16 (VP64) activator domain with D832A dCpf1 to make the dCpf1-VP64 fusion protein (Fig. 4a). To test whether endogenous genes could be activated with this construct, we co-transfected a plasmid harboring this sequence with plasmids encoding crRNAs, caRNAs, and catRNAs targeting DNMT1 and VEGFa promoters into 293T cells. We failed to detect any gene upregulation with crRNAs or caRNAs with either the Pol-II or Pol-III promoter. However, we observed significant gene upregulation with 2 of the 3 catRNAs targeting the DNMT1 promoter and with all 3 of the catRNAs targeting the VEGFa promoter (Fig. 4b).

To test whether a synergistic effect on gene activation exists using different catRNAs simultaneously targeting a promoter, we co-transfected the dCpf1-VP64 fusion protein with pooled crRNAs, caRNAs, catRNAs, and arrayed crRNAs, caRNAs, catRNAs. Although single crRNA and caRNA failed to induce DNMT1 activation, pooled Pol-III and Pol-II caRNAs upregulated gene expression by 2.1-fold and 2.9-fold, respectively. However, no significant gene activation was observed with the pooled crRNAs or the crRNA array, and the highest gene activations were observed with the pooled Pol-II catRNAs (5.3-fold) and the Pol-II array (3.9-fold). No significant difference in gene activation was observed between pooled catRNAs and the catRNA array, and the catRNA array showed better gene activation than the caRNA array. A similar synergistic effect on VEGFa activation was observed with multiple catRNAs and the catRNA array (Fig. 4b).

Because Pol-II crRNAs were not functional and Pol-II caRNAs and Pol-II catRNAs were always more efficient than Pol-III caRNAs and Pol-III catRNAs, we used Pol-III crRNAs, Pol-II caRNAs, and Pol-II catRNAs in future experiments.

To extend the utility of dCpf1 activators, we tethered dCpf1 to a strong synthetic VPR activator, which comprised the VP64 activator, the human NF-KB p65 activation domain, and the Epstein-Barr virus-derived R transactivator (Rta)^30^. dCpf1-VPR led to robust transcriptional upregulation from both of the target gene promoters. Consistent with previous observations with dCas9 activators, gene activation by the dCpf1 activator was more efficient when multiple strong activator domains were recruited to the targeted promoter. Fusion of dCpf1-VPR showed superior activity relative to that of dCpf1-VP64 (Fig. 4c).

dCpf1-VPR induced robust gene activation even with single crRNAs, caRNAs, and catRNAs. Interestingly, crRNAs and caRNAs failed at particular sites, while catRNAs facilitated robust DNMT1 and VEGFa activation at all target sites (Fig. 4c). Furthermore, activation induced by catRNAs was 1.5-fold to 32-fold higher than that induced by crRNAs, further supporting the advantage of using catRNAs for gene perturbation.

### Efficient simultaneous gene activation with the catRNA array

**Fig. 5:**
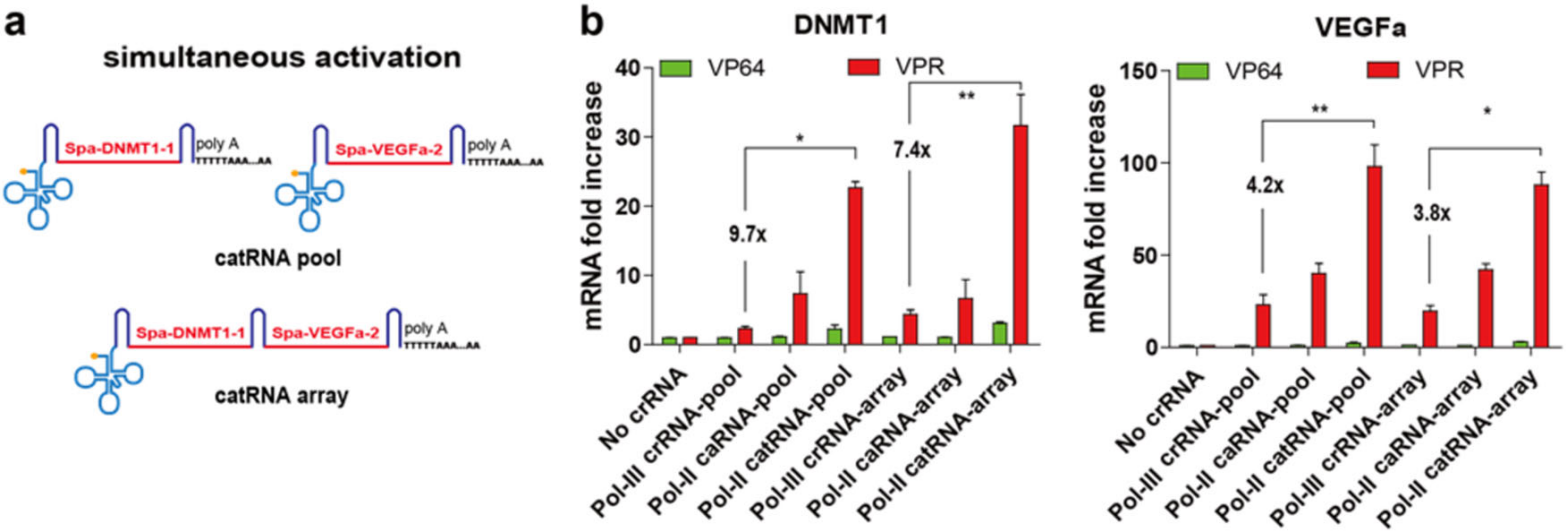
Efficient simultaneous gene activation with a catRNA array. **a** Schematic representation of a catRNA pool and array used for simultaneous gene activation. The spacer sequences used are indicated. **b** Simultaneous DNMT1 and VEGFa gene activation with the dCpf1-VPR activator and catRNA array. Plasmids encoding pooled crRNA, caRNA, and catRNA and arrayed crRNA, caRNA, catRNA were co-transfected with the dCpf1-VPR activator plasmid. Gene expression was measured by quantitative real-time PCR 48 h post-transfection. Bar, SE. *n* = 3. *P < 0.05, **P < 0.01, ***P < 0.001, ****P < 0.0001 determined by the Mann–Whitney test

The capability of processing multiple crRNAs within a single transcript makes Cpf1 a good platform for multiple gene regulation. To test this, we tested a constructed crRNA array targeting the DNMT1 and VEGFa loci (Fig. 5a). We observed simultaneous activation for both endogenous gene promoters with dCpf1-VPR, which had a much higher effect than dCpf1-VP64. In addition, catRNA allowed robust multi-locus activation, as it exhibited expression levels several fold higher than those induced by crRNA and caRNA from both DNMT1 and VEGFa promoters (Fig. 5b). These results suggested that the Cpf1-VPR fusion protein together with the catRNA array enables the simplified and simultaneous activation of multiple genes.

## Discussion

Despite extensive studies on utilizing Cpf1 for gene disruption in mammalian cells and plants, its utility as a gene regulation platform has scarcely been investigated. In this study, we demonstrated that a caged truncated pre-tRNA-like crRNA enables precise and efficient gene ablation with an RNase-resistant property. Moreover, we showed that catRNA enabled efficient gene regulation with dCpf1 activators, while conventional crRNA induced little or no effect. The catRNA design also has profound applications in the gene editing field in embryos and immune cells, such as T cells, wherein RNA electroporation is proven to be more efficient and less toxic than other gene delivery methods^3,31–33^. Although Pol II-transcribed catRNAs with ATG bases in their spacer sequences might potentially be recognized as mRNAs by ribozymes for protein synthesis and affect their gene editing function, we did not observe any side effects of this possible phenomenon, as the catRNAs bearing “ATG” in their spacer sequences used in this study all exhibited efficient DNMT1 and GRIN2b gene disruption. We propose that this was due to catRNA secondary hairpin structures blocking access of the preinitiation complex to mRNA-like catRNA and conferring resistance to helicase unwinding^34–36^. Although we did not observe any evidence that ATG bases in the spacer sequence abolished the function of catRNAs, further studies are needed to fully address this question.

Our finding can improve the utility of Cpf1 in numerous ways. First, enhanced gene disruption may facilitate specific gene knock-ins with catRNA. Second, the improved stability of catRNA enables gene editing with RNA electroporation, wherein crRNA is directly exposed to RNases. Finally, catRNA may facilitate gene regulation with dCpf1 effectors when stable crRNA persistence is required.

One advantage of Cpf1 is the small size of its crRNA, thus leading to a crRNA array for multiplex gene targeting. Although a single catRNA is larger than a crRNA, the catRNA array is a crRNA array embedded in the truncated pre-tRNA structure and not several catRNAs linked together. Thus, the catRNA array is not substantially longer than crRNA array and does not increase the complexity in the array construction.

Although many studies reported the application of the CRISPR/Cpf1 system for specific gene knock-in^9,37,38^, a more recent discovery found that Cpf1 is capable of processing ssDNA, which may explain the extremely low knock-in efficiency of the CRISPR/Cpf1 system where ssDNA is usually used as a donor template^39,40^. Interestingly, although conventional crRNA does not lead to efficient gene knock-in, usage of catRNA still results efficient gene knock-in in the setting of ssDNA degradation by Cpf1.

The lengths of the leader sequence, acceptor stem, T and D domains, and 3′ trailers of a pre-tRNA play important roles in the regulation of tRNA processing and RNase resistance. Further shortening, elongation, nucleotide changes, and modifications on these structures may be implemented to improve the stability and function of catRNA.

In this study, we demonstrated that Cpf1 can be reprogrammed into a gene modulator by harnessing the catalytically dead Cpf1 (dCpf1). Using Pol-II catRNA, we demonstrated that dCpf1 was reprogrammable into a gene activator by fusing with VP64 or VPR activator domains. During preparation of this manuscript, a study reporting reprograming dCpf1 into a gene activator in mammalian cells was published^41^. In their study, the authors failed to induce gene activation with the dCpf1-p65 activator and conventional crRNA, but they later showed that the drug-regulated dCpf1-p65 activator led to transcriptional upregulation. This phenomenon is quite similar to the effect of the dCpf1-VP64 activator in our study, which failed to activate gene expression with conventional crRNA but induced efficient gene activation with catRNA.

A recent study in *Arabidopsis* demonstrated that Cpf1 can be reprogrammed into a gene repressor by fusing an SRDX repressor domain to dCpf1^7^, which is consistent with discoveries in *Eubacterium eligens*^21^ and *Escherichia coli*^22^ demonstrating a reprogrammable feature of Cpf1 for use in gene repression. The precise gene editing feature of Cpf1 makes it a good alternative gene editing candidate to Cas9. The application of dCpf1 as an effector holds great promise in the field when precise gene silencing, rather than gene ablation, is needed. For example, depletion of genes essential for survival may cause early lethality of embryo development, which may be compensated by gene silencing with dCas9 or dCpf1-KRAB suppressors. In addition, cell fate reprogramming with dCpf1 gene activators also hold great promise in the stem cell biology and regenerative medicine fields.

A very recent study demonstrated that Pol-II-transcribed crRNAs exert higher gene disruption efficiencies than Pol-III-transcribed crRNAs^27^, perhaps due to elevated crRNA expression and tandem direct repeats rather than RNase-resistance provided by the combination of truncated pre-tRNAs, 5′ caps and 3′ tails of catRNAs. In this study, substantially increased gene targeting largely resulted from the additive effect of the truncated pre-tRNA, 5′ cap and 3′ tail rather than any combination of two elements used.

In conclusion, we demonstrated that genetic editing and interrogation in mammalian cells can be accomplished with Cpf1 and caged truncated pre-tRNA-like crRNAs.

## Materials and methods

### Plasmid construction

D832A-mutated DNA endonuclease-dead LbCpf1 was cloned into the pcDNA3.1 vector based on the sequence provided by Addgene. Different activator domains were fused to dCpf1 using a nuclear localization signal and a (GS)n linker. CrRNAs, caRNAs, and catRNAs were cloned into pcDNA3.1 vectors driven by U6, T7, or EF1a promoters. To initiate efficient transcription under the U6 and T7 promoters, a single “G” nucleotide was added in front of all the crRNA, caRNA, and catRNA sequences.

### Plasmid DNA transfection

HEK293T cells (ATCC) were cultured in DMEM (Life Technologies, Carlsbad, CA, USA) supplemented with 10% FBS (Omega Scientific) and 25 mM Hepes. Cells were transfected in 6-well plates at 70% confluency with 2 μg of plasmid encoding LbCpf1 and 2 μg of plasmid encoding crRNA (caRNA, catRNA) using Lipofectamine 3000 (Life Technologies). Cells were harvested for genomic DNA extraction 72 h after DNA transfection.

### In vitro transcription (IVT)

For electroporation, LbCpf1 mRNA and different Pol-II crRNA species were transcribed in vitro using mMESSAGE mMACHINE T7 ULTRA kits (Life Technologies, AM1345) with capping and tailing. Different Pol-III crRNA species were transcribed using a MEGAscript T7 Transcription Kit (AM1334, Life Technologies, Carlsbad, CA). Sequences for crRNA, caRNA, catRNA, and full-length pre-tRNA-crRNA for each experiment are listed in Supplementary Table 1.

### RNA transfection

Cells were transfected in 6-well plates at 70% confluency with 2 μg of Cpf1 mRNA and 2 μg of crRNA (caRNA, catRNA) using the TransIT^®^-mRNA Transfection kit (Mirus). Cells were harvested for flow cytometry analysis and genomic DNA and RNA extraction 48 h after RNA transfection.

### Electroporation

Briefly, 293T cells were trypsinized and harvested in growth medium supplemented with serum. The cells were washed with Opti-MEM two times and then resuspended in Opti-MEM at a final concentration of 1 × 10^7^ cells/ml. Subsequently, the cells (0.1 ml) were mixed with IVT RNA and electroporated in a 2-mm cuvette. Briefly, 20 μg of LbCpf1 mRNA together with 20 μg of crRNA (caRNA, catRNA) were electroporated into the cells using a BTX830 electroporator (Harvard Apparatus BTX) at 200V for 5 ms. Following electroporation, the cells were immediately placed in 2 ml of pre-warmed antibiotic-free culture medium and incubated at 37 °C and 5% CO_2_.

### Real-time PCR

Total RNA was isolated using the RNeasy Plus RNA isolation kit (Qiagen), and cDNA synthesis was performed using the SuperScript VILO cDNA synthesis kit (Invitrogen). Real-time PCR using PerfeCTa SYBR Green FastMix was performed on the CFX96 Real-Time PCR Detection System (Bio-Rad) with the oligonucleotide primers shown in Supplementary Table 2. Primer specificity was confirmed by agarose gel electrophoresis and melting curve analysis. The results are expressed as the fold increase in mRNA expression of the gene of interest normalized to that of GAPDH. The values are reported as the mean ± s.e.m. from three independent experiments performed on different days (*n* = 3) with technical duplicates that were averaged for each experiment.

### In vitro RNA digestion

First, 1 μg of in vitro transcribed Pol III and Pol II-like crRNA, caRNA, and catRNA was incubated with 0.1 μg RNase (Thermo Fisher) for 30 min. Then, RNA was separated on denaturing 10% polyacrylamide gels (8 M urea, 1× TBE) and transferred by semi-dry blotting on a nylon membrane (Hybond TM N+, GE Healthcare). Chemical crosslinking was performed for 1 h at 60 °C with 1-ethyl-3-(3-dimethylaminopropyl) carbodiimide hydrochloride. Oligonucleotides were radioactively labeled with [γ-32P] ATP (5000Ci mmol−1) and T4 polynucleotide kinase (Fermentas) and purified using Illustra Microspin G-25 columns (GE Healthcare). The hybridization of the probe against the spacer in the crRNA (Supplementary Table 1) was performed in Rapid-hyb buffer (GE Healthcare) by incubation overnight at 42 °C. The radioactive signal was visualized using phosphorimaging.

### Data availability

All data supporting the findings of this study are included in the manuscript and its Supplementary Files or are available from the authors upon request.

## Electronic supplementary material

Supplementary data

Supplementary data
