# Supplementary material for: Genetic editing and interrogation with Cpf1 and caged truncated pre-tRNA-like crRNA in mammalian cells
Source: Cell Discov. 2018 Jul 10;4:36. doi: 10.1038/s41421-018-0035-0 (PMC6037731; doi:10.1038/s41421-018-0035-0)
Supplement: Supplementary file 1 — Supplementary data [file 41421_2018_35_MOESM1_ESM.docx]

**SUPPLEMENTARY DATA**


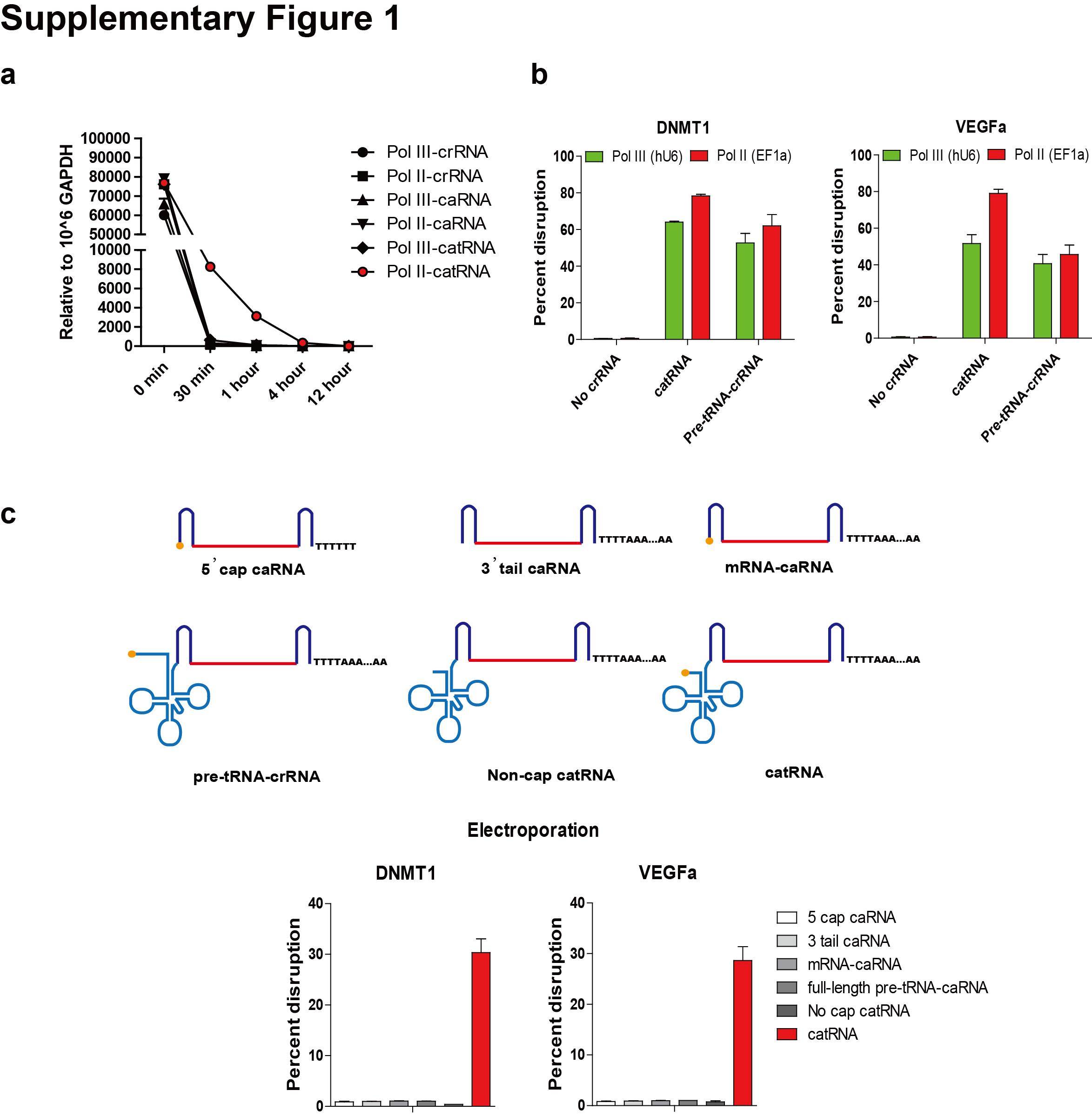


**Supplementary Figure 1. Enhanced stability of RNase-resistant catRNA.**

a. Stabilities of crRNA, caRNA and catRNA as measured by real-time PCR. Pol-II and Pol-III crRNAs, caRNAs and catRNAs were electroporated into 293T cells. Total RNAs isolated from 293T cells at 0 min, 30 min, 1 hour, 4 hours and 12 hours after electroporation were subjected to real-time PCR analysis to detect the persistence of the crRNA species. b. Gene disruption by full-length pre-tRNA-caRNA and truncated pre-tRNA-caRNA (catRNA) using the DNA transfection method. c. Gene disruption by various modified crRNAs using the RNA electroporation method. Gene disruption efficiency was calculated 3 days after transfection with TIDE software. Bar, SE. n=3.


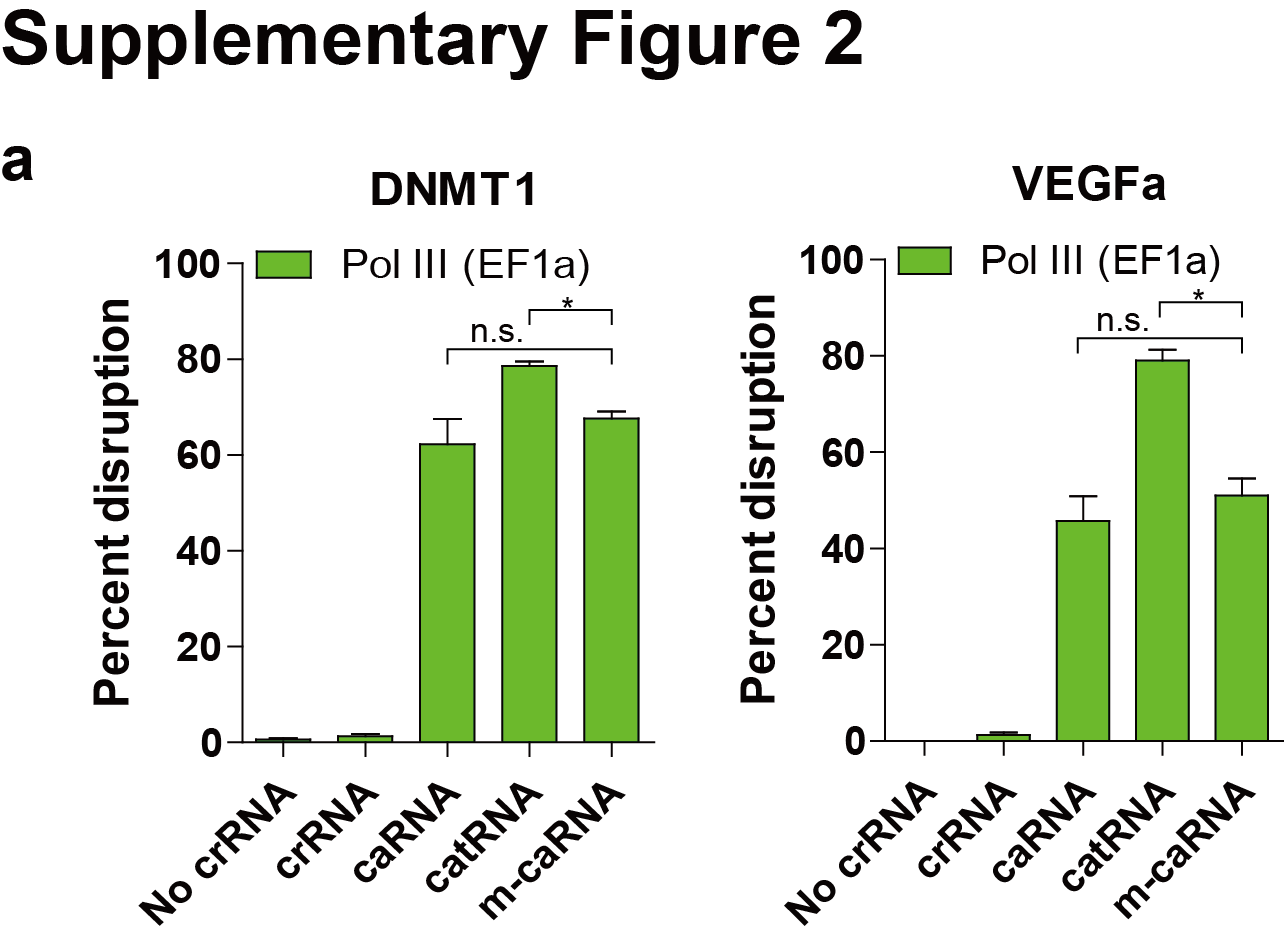


**Supplementary Figure 2. m-caRNA displays a gene targeting efficiency equal to that of caRNA but less than that of catRNA.**

a. Targeted gene knock-out with crRNA, caRNA, catRNA and m-caRNA in 293T cells. Gene knock-out was performed by co-transfecting the Cpf1 DNA plasmid and plasmids encoding the crRNA species with Lipofectamine 3000. Specific gene knock-outs were measured 3 days after transfection with TIDE online software. Bar, SE. n=3. *, P<0.05, **, P<0.01, ***, P<0.001, ****, P<0.0001 determined by the Mann-Whitney test.

**
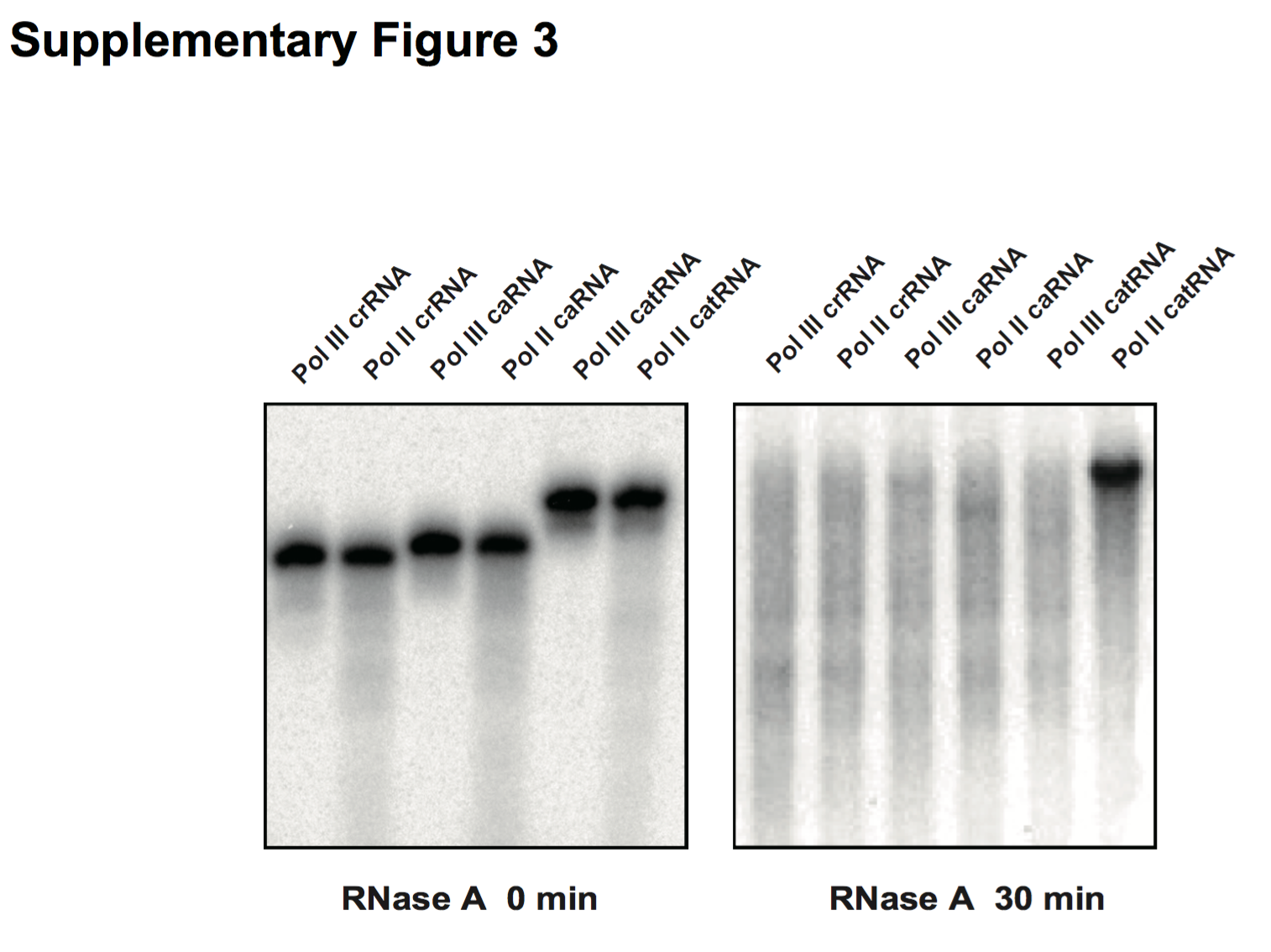
**

**Supplementary Figure 3. In vitro digestion of crRNA species by RNase A.**

In vitro digestion was performed by incubation of RNase A with catRNA, caRNA and crRNA transcribed by either Pol II or Pol III. RNA stability was measured by northern blot before and 30 min after incubation.


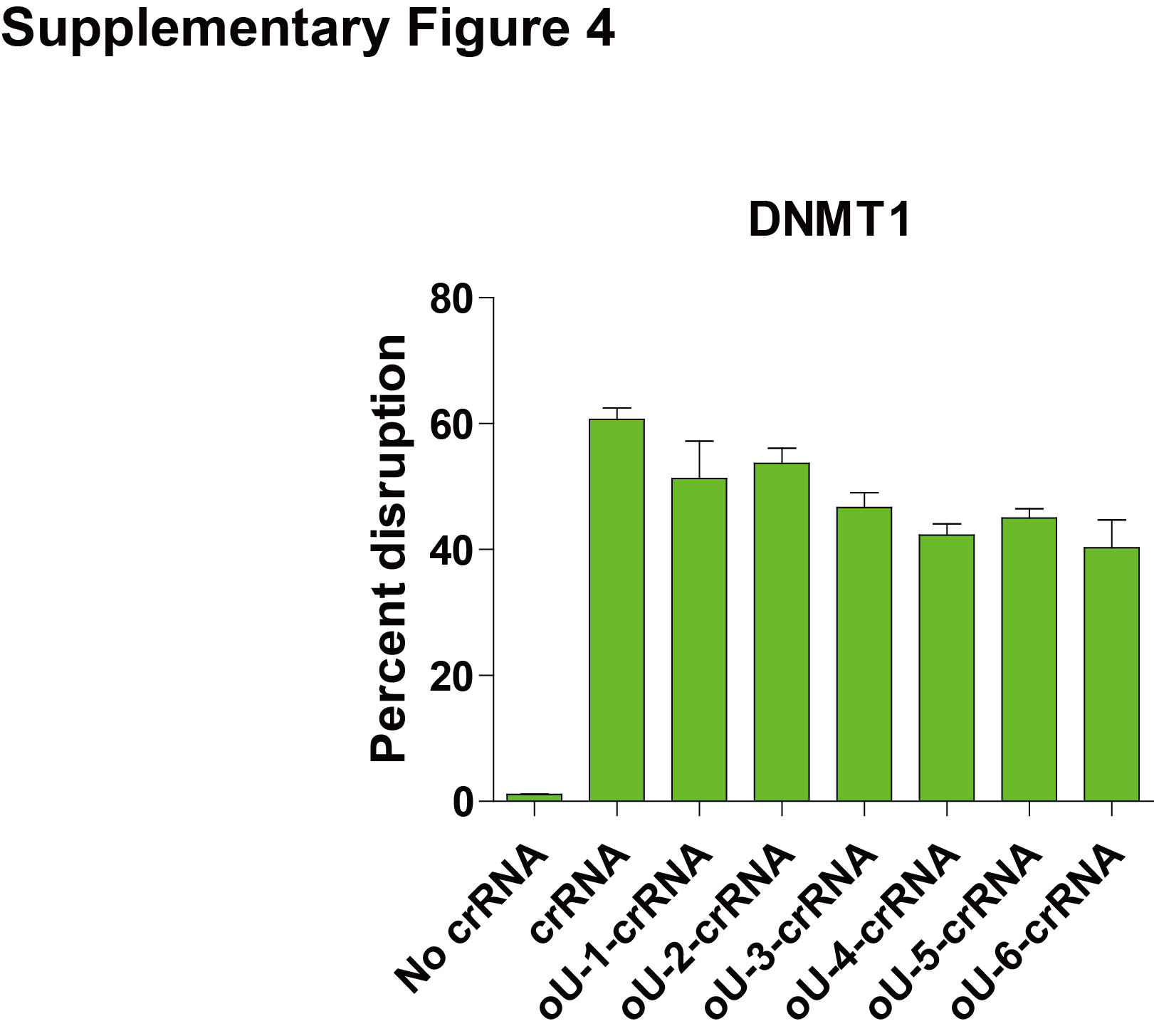


**Supplementary Figure 4. Mismatched oligo-U in crRNA reduces the targeting efficiency.**

Conventional crRNA and crRNA with different 3’ terminal oligo U sequences (oU-crRNAs) targeting DNMT1 and VEGFa genes were transcribed in vitro by T7 polymerase. The gene disruption efficiency of crRNA and oU-crRNA were compared by electroporating equal amount of crRNA and oU-crRNA after Cpf1 mRNA electroporation into 293T cells. PCR amplicons flanking the target site were amplified from genomic DNA isolated 3 days after transfection. The levels of genomic disruption of DNMT1 with different crRNA species were determined with TIDE online software. The numbers in the oU-crRNAs indicates the number of oligo-T in the crRNA sequence. Bar, SE. n=3.


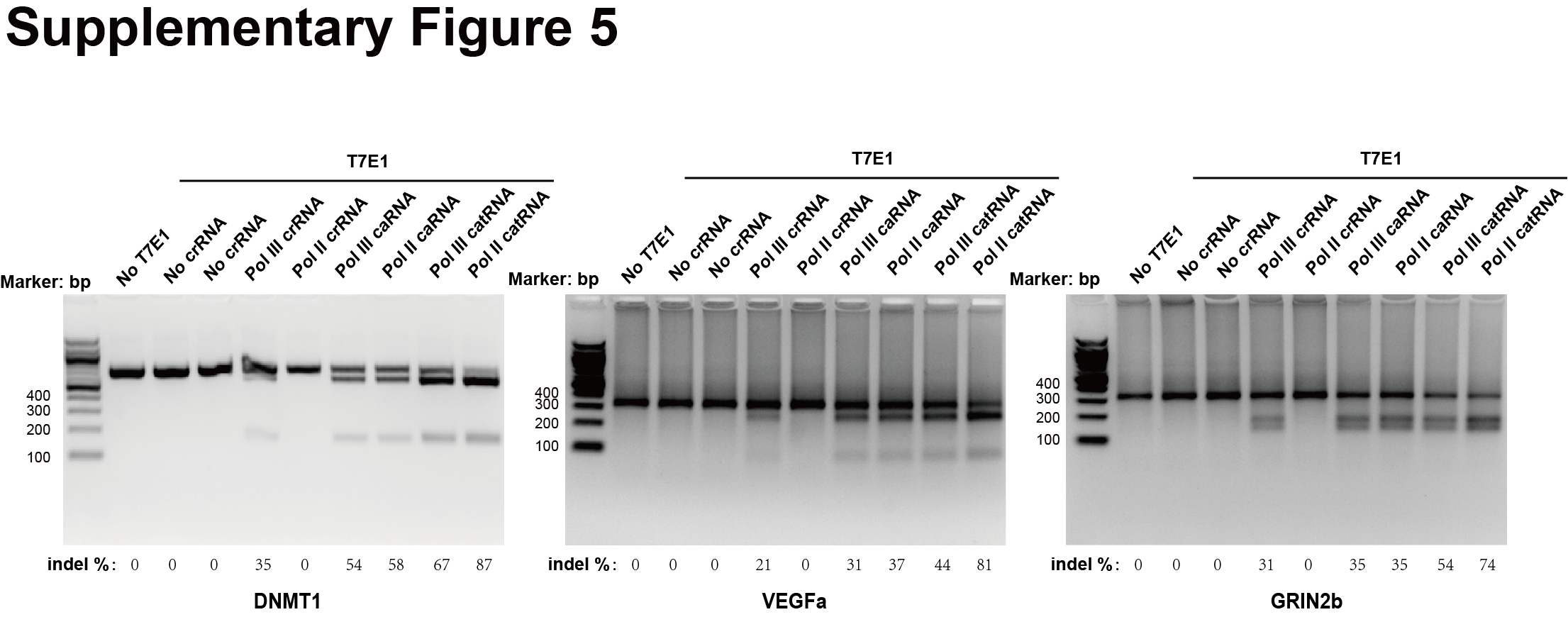


**Supplementary Figure 5. catRNA enhances targeted knock-out in mammalian cells.**

Gene knock-out was performed by co-transfecting the LbCpf1 DNA plasmid and plasmids encoding different crRNA species with Lipofectamine 3000. PCR amplicons flanking the target site were amplified from genomic DNA isolated 3 days after transfection. The levels of genomic disruption of DNMT1, VEGFa, and GRIN2b with different crRNA species were determined by a T7E1 nuclease assay (NEB). The percent target disruption was quantified by densitometry.


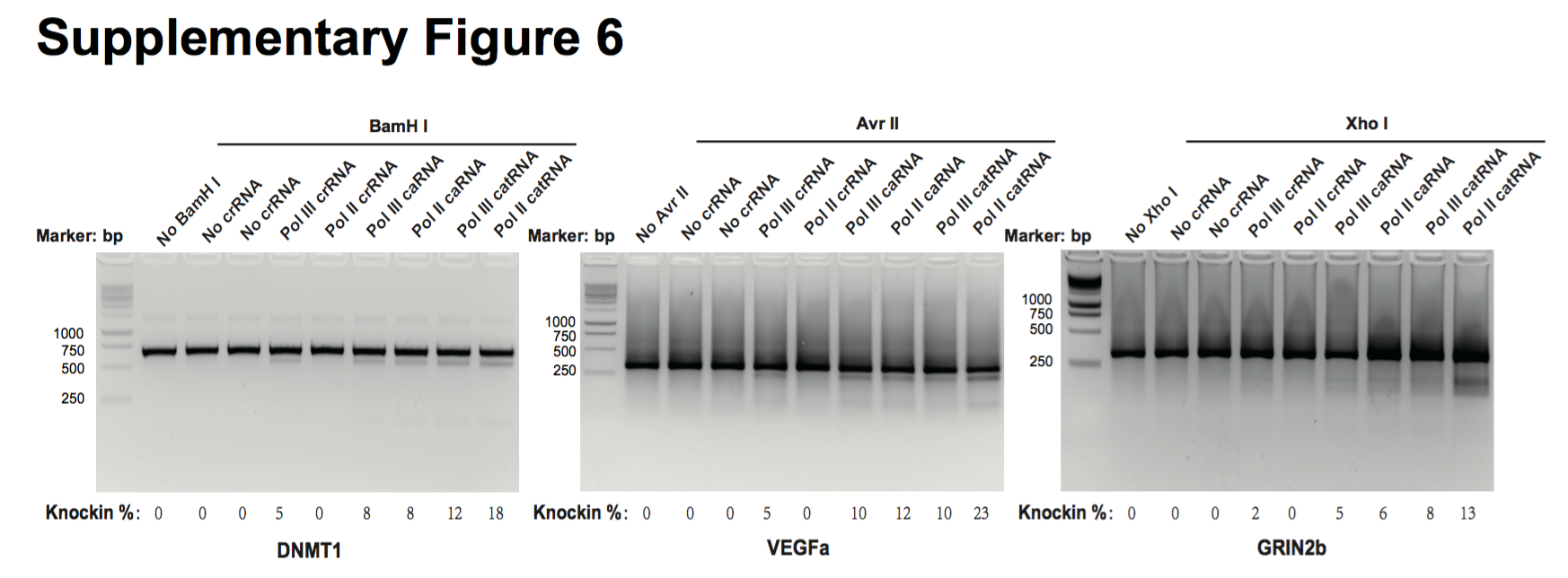


**Supplementary Figure 6. catRNA enhances targeted knock-in in mammalian cells.**

To test the gene knock-in level with methods other than TIDE, new restriction enzymatic sites were introduced into DNMT1, VEGFa, and GRIN2b loci with different ssDNA donor templates harboring these restriction enzymatic sites. PCR amplicons flanking the target site were amplified from genomic DNA isolated 3 days after transfection. Enzymatic digestion was performed to verify the knock-in efficiency for each gene.
