# Supplementary material for: Genetic editing and interrogation with Cpf1 and caged truncated pre-tRNA-like crRNA in mammalian cells
Source: Cell Discov. 2018 Jul 10;4:36. doi: 10.1038/s41421-018-0035-0 (PMC6037731; doi:10.1038/s41421-018-0035-0)
Supplement: Supplementary file 2 — Supplementary data [file 41421_2018_35_MOESM2_ESM.docx]

**Exp-2，3**

**Full-length guide RNA sequences:**

DNMT1-crRNA GAATTTCTACTAAGTGTAGATCTGATGGTCCATGTCTGTTACTCTTTTTT

VEGFa-crRNA GAATTTCTACTAAGTGTAGATCTAGGAATATTGAAGGGGGCAGGTTTTTT

GRIN2b-crRNA GAATTTCTACTAAGTGTAGATGTGCTCAATGAAAGGAGATAAGGTTTTTT

DNMT1-caRNA GAATTTCTACTAAGTGTAGATCTGATGGTCCATGTCTGTTACTCAATTTCTACTAAGTGTAGATTTTTTTAAAAAA

VEGFa-caRNA GAATTTCTACTAAGTGTAGATCTAGGAATATTGAAGGGGGCAGGAATTTCTACTAAGTGTAGATTTTTTTAAAAAA

GRIN2b-caRNA GAATTTCTACTAAGTGTAGATGTGCTCAATGAAAGGAGATAAGGAATTTCTACTAAGTGTAGATTTTTTTAAAAAA

DNMT1-catRNA GCCAGTGGTCTAGTGGTAGAATAGTACCCTGCCACGGTACAGACCCGGGTTCGATTCCCGGCTGGAAATAATTTCTACTAAGTGTAGATCTGATGGTCCATGTCTGTTACTCAATTTCTACTAAGTGTAGATTTTTTTAAAAAA

VEGFa-catRNA GCCAGTGGTCTAGTGGTAGAATAGTACCCTGCCACGGTACAGACCCGGGTTCGATTCCCGGCTGGAAATAATTTCTACTAAGTGTAGATCTAGGAATATTGAAGGGGGCAGGAATTTCTACTAAGTGTAGATTTTTTTAAAAAA

GRIN2b-catRNA GCCAGTGGTCTAGTGGTAGAATAGTACCCTGCCACGGTACAGACCCGGGTTCGATTCCCGGCTGGAAATAATTTCTACTAAGTGTAGATGTGCTCAATGAAAGGAGATAAGGAATTTCTACTAAGTGTAGATTTTTTTAAAAAA

**Full-length pre-tRNA-crRNA guide RNA sequences**:

DNMT1-pre-tRNA-crRNA GAACAAAGCACCAGTGGTCTAGTGGTAGAATAGTACCCTGCCACGGTACAGACCCGGGTTCGATTCCCGGCTGGTGCAAAATAATTTCTACTAAGTGTAGATCTGATGGTCCATGTCTGTTACTCAATTTCTACTAAGTGTAGATTTTTTTAAAAAA

VEGFa-pre-tRNA-crRNA GAACAAAGCACCAGTGGTCTAGTGGTAGAATAGTACCCTGCCACGGTACAGACCCGGGTTCGATTCCCGGCTGGTGCAAAATAATTTCTACTAAGTGTAGATCTAGGAATATTGAAGGGGGCAGGAATTTCTACTAAGTGTAGATTTTTTTAAAAAA

GRIN2b-pre-tRNA-crRNA GAACAAAGCACCAGTGGTCTAGTGGTAGAATAGTACCCTGCCACGGTACAGACCCGGGTTCGATTCCCGGCTGGTGCAAAATAATTTCTACTAAGTGTAGATGTGCTCAATGAAAGGAGATAAGGAATTTCTACTAAGTGTAGATTTTTTTAAAAAA

**Primers for TIDE**

DNMT1-f AAGGATCTTGTGCTGGAAGGGTTTT

DNMT1-r AATCCAGAATGCACAAAGTACTGCACA

VEGRa-f GAGCCAGCCCTTTTCCTCATAAGG

VEGFa-r GCGTGATGATTCAAACCTACCCG

GRIN2b-f AAAACAGTATGGGGGAGAACACGTATT

GRIN2b-r GGAACTGGTAGCCATGAATGAGACC

**Primers for real-time PCR:**

DNMT1-f CGTCCCTATGCTGGTTCCTTACTGT

DNMT1-r GGGAGGGCAGAACTAGTCCTTAGCA

VEGRa-f TTGGCATTTTACTTCAATGTGCCTCA

VEGFa-r CAGAGGTAGCCAAGAGCCCCAAAC

**ssDNA donor templates:**

DNMT1-ssDNA-donor CGGTGTCACGCCACTTGACAGGCGAGTAACAGACATGGACCATCAGGTAACATTAACGTACTGATGTTAACAGCTGACCCAAT

VEGFa-ssDNA-donor CGTCCCTGTCCGGCTCTCCGCCTTCCCCTGCCCCCTTCAATATTCCTAGCTAAGAGGGAACGGCTCTCAGGCCCTGTCCG

GRIN2b-ssDNA-donor AGAGGCTAGATACTGCAATTCAAGGACCTTATCTCCTTTCATTGAGCACCTAACCCAACTCCATCTACCAGCCTACTCTCTTATCT

**Exp-4**

**23-nt guide RNA spacer sequences:**

DNMT1-crRNA-1 TCAGCACCATTTGTTAAAGACAC

DNMT1-crRNA-2 CGCGCGAAAAGCCGGGGCGCCTG

DNMT1-crRNA-3 TGAGAGCCCTTGAGTAAAGTCCT

VEGFa-crRNA-1 TGACCTCCCAAACAGCTACATAT

VEGFa-crRNA-2 CTGCTCCCTCCTCGCCAATGCCC

VEGFa-crRNA-3 TCCCCAAATCACTGTGGATTTTG

**Exp-5**

**23-nt guide RNA spacer sequences:**

DNMT1-crRNA-1 TCAGCACCATTTGTTAAAGACAC

VEGFa-crRNA-2 CTGCTCCCTCCTCGCCAATGCCC

**Supplementary Figure 1**

**Primers for real-time PCR:**

DNMT1-crRNA-f1 GAATTTCTACTAAGTGTAGATCTGATGG

DNMT1-crRNA-r1 AAAAAAGAGTAACAGACATGGACC

VEGFa-crRNA-f1 GAATTTCTACTAAGTGTAGATCTAGGAAT

VEGFa-crRNA-r1 AAAAAACCTGCCCCCTTCAA

GRIN2b-crRNA-f1 GAATTTCTACTAAGTGTAGATGTGCT

GRIN2b-crRNA-r1 AAAAAACCTTATCTCCTTTCATTGAGC

DNMT1-caRNA-f1 GTAGATCTGATGGTCCATGTCTGTT

DNMT1-caRNA-r1 AAAAAAATCTACACTTAGTAGAAATTGAGTAA

VEGFa-caRNA-f1 GTAGATCTAGGAATATTGAAGGGGG

VEGFa-caRNA-r1 AAAAAAATCTACACTTAGTAGAAATTCCTG

GRIN2b-caRNA-f1 GTAGATGTGCTCAATGAAAGGAGAT

GRIN2b-caRNA-r1 AAAAAAATCTACACTTAGTAGAAATTCCTTAT

catRNA-f1 GCCAGTGGTCTAGTGGTAGAATAGT

DNMT1-catRNA-r1 GAGTAACAGACATGGACCATCAGAT

VEGFa-catRNA-r1 TGCCCCCTTCAATATTCCTAGAT

GRIN2b-catRNA-r1 CCTTATCTCCTTTCATTGAGCACAT

**Supplementary Figure 3**

**Probe for Northern blot of DNMT1 crRNAs**

GAGTAACAGACATGGACCATCAGATCTACA

**Supplementary Figure 5,6**

**Primers for PCR:**

DNMT1-f TTGAGCCTCTGGGTCTAGAACCCTC

DNMT1-r TCCATTTGCATGTCATGGGGATC

VEGRa-f AATAGGGGGTCCAGGAGCAAACTC

VEGFa-r AGCAGGAAAGTGAGGTTACGTGCG

GRIN2b-f AACAGCACTCCGCTCTGGGCTT

GRIN2b-r CTGCCTGTAGCTGCCAATGACTATAGC

**ssDNA donor templates:**

DNMT1-ssDNA-donor-2 CGGTGTCACGCCACTTGACAGGCGAGTAACAGACATGGACCATCAggatccATTAACGTACTGATGTTAACAGCTGACCCAAT

VEGFa-ssDNA-donor-2

CGTCCCTGTCCGGCTCTCCGCCTTCCCCTGCCCCCTTCAATATTcctaggTAAGAGGGAACGGCTCTCAGGCCCTGTCCG

GRIN2b-ssDNA-donor-2

AGAGGCTAGATACTGCAATTCAAGGACCTTATCTCCTTTCATTgagctcCTAACCCAACTCCATCTACCAGCCTACTCTCTTATCT
